# Supplementary material for: All-Trans Retinoic Acid Effect on Candida albicans Growth and Biofilm Formation
Source: J Fungi (Basel). 2022 Oct 5;8(10):1049. doi: 10.3390/jof8101049 (PMC9605275; doi:10.3390/jof8101049)
Supplement: Supplementary file 1 [file jof-08-01049-s001.zip › jof-1900227-supplementary.pdf]

Table S1. Effect of ATRA on biomass and metabolic activity of *C. albicans* biofilm. *Candida* cultures were incubated for 24 h in the absence or presence of ATRA at various concentrations (1- 0.06 mM). Biofilm growth was analyzed as total biomass and metabolic activity by CV and XTT reduction methods, respectively. The absorbance intensity was measured using a spectrophotometer plate reader at 595 nm for CV and 490 nm for XTT assay. The results of mean OD ± SD of three independent experiments, carried out in triplicate, have been reported. AmB (2- 0.12 μg/mL) was used as positive control drug.

		mean value OD	SD	% of inhibition
**Biomass at baseline**	ATRA 1 mM	0.082	0.036	
	ATRA 0.5 mM	0.086	0.032	
	AmB 2μg/mL	0.073	0.002	
	AmB 1μg/mL	0.079	0.002	
**Biofilm biomass after 24 h**	Control	0.65	0.014	
	ATRA 1mM	0.064	0.006	90
	ATRA 0.5mM	0.091	0.003	86
	ATRA 0.25mM	0.153	0.052	76
	ATRA 0.12mM	0.316	0.019	50
	ATRA 0.06mM	0.59	0.011	5
	AmB 2μg/mL	0.071	0.008	90
	AmB 1μg/mL	0.076	0.008	89
	AmB 0.5μg/mL	0.084	0.012	87
	AmB 0.25μg/mL	0.176	0.035	73
	AmB 0.12μg/mL	0.493	0.023	25
**Biofilm metabolic activity after 24 h**	Control	0.62	0.031	
	ATRA 1mM	0.064	0.002	90
	ATRA 0.5mM	0.091	0.001	86
	ATRA 0.25mM	0.26	0.005	59
	ATRA 0.12mM	0.443	0.047	29
	ATRA 0.06mM	0.605	0.044	3
	AmB 2 μg/mL	0.07	0.035	89
	AmB 1 μg/mL	0.112	0.031	82
	AmB 0.5 μg/mL	0.20	0.006	68
	AmB 0.25 μg/mL	0.333	0.031	47
	AmB 0.12 μg/mL	0.544	0.033	9

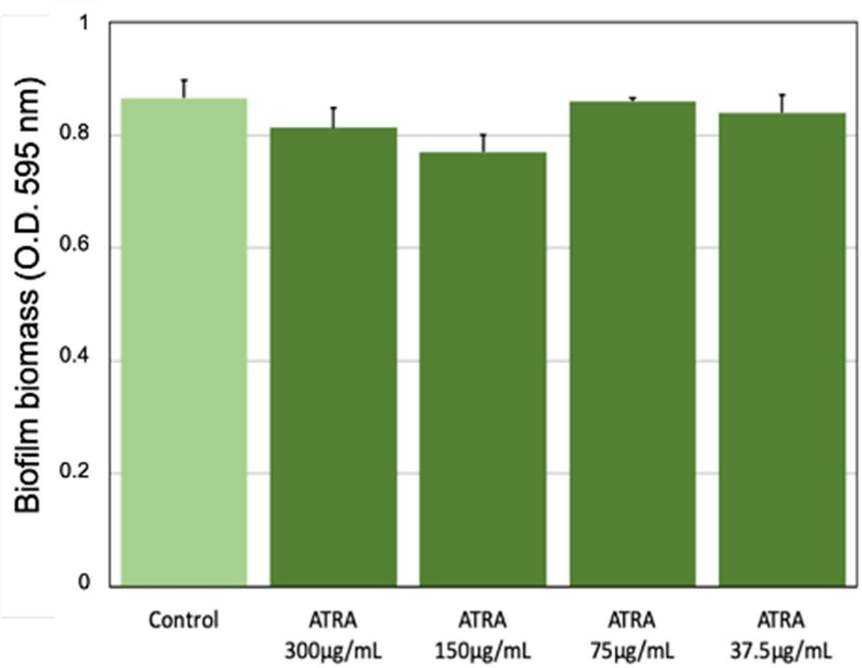

Figure S1. Effect of ATRA on preformed biofilm of *C. albicans*. 24-h old *C. albicans* biofilms were treated with ATRA at various concentrations (300 - 37.5 µg/mL) and further incubated for 24 h. Biofilm growth was analyzed as total biomass by crystal violet assay. The absorbance intensity of the crystal violet dye was measured using a spectrophotometer plate reader at 595 nm. Results are the means ± SD of three independent experiments carried out in triplicate.
